# Lectin from Sambucus sieboldiana abrogates the anoikis resistance of colon cancer cells conferred by N-acetylglucosaminyltransferase V during hematogenous metastasis

**DOI:** 10.18632/oncotarget.15034

**Published:** 2017-02-02

**Authors:** Kyoung Jin Song, Seong Kook Jeon, Su Bin Moon, Jin Suk Park, Jang Seong Kim, Jeongkwon Kim, Sumin Kim, Hyun Joo An, Jeong-Heon Ko, Yong-Sam Kim

**Affiliations:** ^1^ Genome Editing Research Center, KRIBB, Daejeon, South Korea; ^2^ Biotherapeutics Translational Research Center, KRIBB, Daejeon, South Korea; ^3^ Department of Chemistry, Chungnam National University, Daejeon, South Korea; ^4^ Graduate School of Analytical Science and Technology, Chungnam National University, Daejeon, South Korea; ^5^ Asia-Pacific Glycomics Reference Site, Daejeon, South Korea; ^6^ Department of Biomolecular Science, Korea University of Science and Technology, Daejeon, South Korea

**Keywords:** anoikis, metastasis, MGAT5, SSA

## Abstract

Anoikis is a form of anchorage-dependent apoptosis, and cancer cells adopt anokis-resistance molecular machinery to conduct metastasis. Here, we report that N-acetylglucosaminyltransferase V gene expression confers anoikis resistance during cancer progression. Overexpression of N-acetylglucosaminyltransferase V protected detached cancer cells from apoptotic death, and suppression or knockout of the gene sensitized cancer cells to the apoptotic death. The gene expression also stimulated anchorage-dependent as well as anchorage-independent colony formation of cancer cells following anoikis stress treatments. Importantly, treatment with the lectin from *Sambucus sieboldiana* significantly sensitized anoikis-induced cancer cell deaths *in vitro* as well as *in vivo*. We propose that the lectin alone or an engineered form could offer a new therapeutic treatment option for cancer patients with advanced tumors.

## INTRODUCTION

Tumor cells acquire an ability to spread to distant locations by equipping themselves with a variety of molecular machinery. The primary goal for a cancer cell would be to penetrate the circulatory system such as lymphatic or blood vessels after local disseminations to facilitate distal metastasis. During local disseminations, circulation inside blood or lymphatic vessels, and ectopic colonization, cancer cells undergo an anchorage-dependent apoptotic and/or necrotic stress, termed anoikis [1]. Except for certain cell types such as blood cells, most cells are inter-connected via cell-cell and cell-extracellular matrix adhesions [2], and it is commonly observed that those cells develop apoptotic and necrotic signatures when placed under detached conditions [3, 4]. The deregulation of anoikis is emerging as a hallmark of cancer [5], and resistance to anoikis stress is one of the critical aspects for cancer stem cells [6] and circulating tumor cells (CTCs) [7]. Epithelial-mesenchymal transition was proposed as one of the mechanism underlying the acquisition of anoikis resistance of cancer cells [8]. Characterization of anoikis-resistant cells identified several important players including αvβ6 integrin [9], PTEN mutation [10], neurotrophic tyrosine kinase receptor B [11], microRNAs [12], etc.; however, there is no systemic approach by which therapeutic options for patients with metastatic behaviors are suggested to control anoikis resistance.

N-acetylglucosaminyltransferase V (MGAT5 or GnT-V) is a golgi-located enzyme that catalyzes the branching of the β1,6-N-acetylglucosamine side chain to the core mannosyl residue of N-linked glycan. MGAT5 expression has been shown to harness cancer metastasis in a mouse model *in vivo* [13], and the overexpression of the MGAT5 gene has been found in various tumor cells and tissues [14–16]. Previously, we showed the involvement of MGAT5 in the stimulation of metastasis through the alteration of the glycosylation of TIMP-1 *in vitro* and *in vivo* [17, 18]. The aberrant TIMP-1 significantly lost gelatinases inhibition, which culminated in an elevated metastatic potential of colon cancer cells. MGAT5-induced regulation of MT1-MMP was also suggested as one of the strategies. MGAT5 stimulated MT1-MMP expression in cancer cells and the elevated MT1-MMP expression enhanced the proteolytic capacity in colon cancer cells, thereby reinforcing their invasive and metastatic potential [19]. Besides involvements in a variety of stages during cancer progression, MGAT5 has been reported to confer anoikis resistance in liver [20] and colon cancer [21]. Nonetheless, more strategic approaches to control MGAT5-mediated anoikis resistance are demanding.

Here, we found through transcriptional profiling that the MGAT5 gene is a key regulator of anoikis resistance in colon cancer, and that the lectin from *Sambucus sieboldiana* (SSA) efficiently sensitized colon cancer cells to anoikis *in vitro* and *in vivo*: MGAT catalyzed the formation of the pro-metastatic a β1,6-GlcNAc linkage of N-glycan, and SSA interfered with the pro-metastatic action of the glycan linkage in cancer cells. Given the importance of the acquisition of anoikis resistance of cancer cells during metastasis, SSA, as itself or an engineered form, could provide an important treatment option for cancer patients, particularly those who are diagnosed with CTCs or those who are likely to have dormant cancer cells in their circulatory system.

## RESULTS

### Transcriptomic analysis identified the MGAT5 gene as a key modulator of anoikis resistance

**Figure 1:**
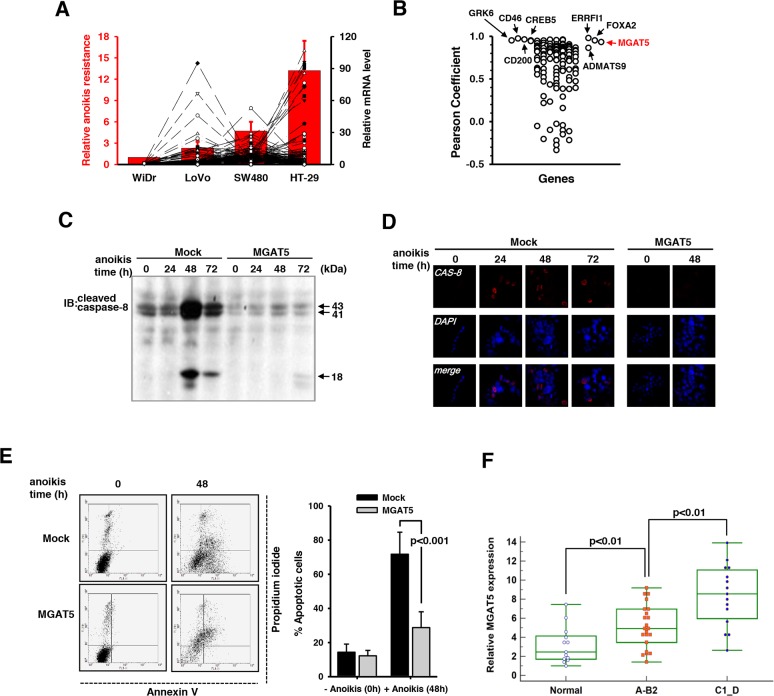
Identification of MGAT5 as a key regulatory gene for anoikis resistance **A**. The relative anoikis resistance of four colon cancer cell lines and the relative transcription levels with a ratio of more than 2, when compared to that of WiDr were plotted (n=5). **B**. Calculation of Pearson correlation coefficient (*r*) identified MGAT5 as a key regulatory gene (*r=*0.932). **C**. Caspase-8 activation in the mock and WiDr:MGAT5 cells was compared by immunoblot analyses using an anti-cleaved caspase-8 antibody. **D**. The anoikis-related death was monitored by immunofluorescence using an anti-cleaved caspase-8 antibody. **E**. The early and late apoptotic deaths were monitored by Annexin V and propidium iodide staining, respectively, using a flow cytometer. The percent apoptotic cells were calculated from the flow cytometer results compared between mock and MGAT5-overexpressing cells (averages with S.E. for n=3). **F**. The MGAT5 transcription levels were measured by quantitative real time-PCR using colon cancer tissues and their normal counterparts. Relative mRNA levels were calculated from the Ct values for MGAT5 normalized by those for GAPDH. Colon cancer stages were divided into a lower Astler-Coller grades (A-B2) and higher ones (C1-D).

**Figure 2:**
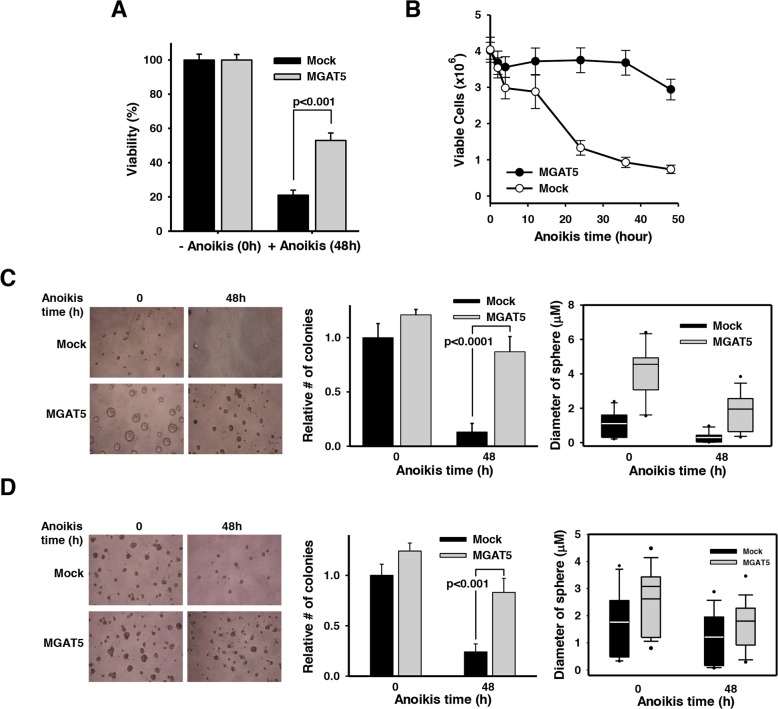
Anchorage-dependent and independent growth advantages by MGAT5-induced anoikis resistance **A**. Cell viability was assessed by an XTT assay to compare the anoikis resistance of mock and WiDr:MGAT5. Cells (1×10^4^) were placed into each well of 96-well plates and incubated in the presence of XTT solution for 2 hours. Absorbance at 450nm was measured in an ELISA reader (n=3). **B**. Cells were treated with anoikis stress for indicated times and allowed to form colonies on a culture plate. The number of colony was converted into viable cells by considering diluting factors (×1,000) (n=5). **C-D**. Cells were treated with anoikis stress for 48 hours and then embedded into soft agar **(C)** and basement membrane-based Matrigel **(D)**. Cells were allowed to grow and the number of colonies and the diameter of colony spheres were measured (n=3).

A systemic identification of regulators for anoikis resistance is necessary to control cancer metastasis and to develop new therapeutics. To search for key regulatory genes involved in anoikis resistance, four colon cancer cell lines with different levels of anoikis resistance including WiDr, LoVo, SW480, and HT-29 were compared in terms of their relative transcription levels and anoikis resistance. Cells were subjected to anoikis stress for 48 hours on agar-coated culture plates, and the viable colonies were counted. This anoikis resistance test revealed that anoikis resistance increased in the order of WiDr, LoVo, SW480, and HT-29 (Figure 1A). Gene expression profiling was conducted by DNA microarray analysis where cells were exposed to anoikis stress for 48 hours and the relative mRNA levels (Lovo vs WiDr, SW480 vs WiDr, and HT-29 vs WiDr) were investigated. The relative values of more than 2 were considered to be significant and to contribute to anoikis resistance. This analysis identified a total of 124 genes (Supplementary Table 1). The Pearson correlation coefficients were calculated for each gene in relation with anoikis resistance and the MGAT5 expression level. Several genes of interests were mined including CD200, GRK6, KRAS, and FOXA2; however, we focused on the MGAT5 gene with a Pearson correlation coefficient of 0.932 (Figure 2B).

To validate the involvement of MGAT5 in the anoikis resistance, a WiDr cell line with stable MGAT5 overexpression (WiDr:MGAT5) was established. Caspase-8 cleavage is a hallmark signature in the anoikis-related pathway [22]; thus, we considered the protein cleavage as a surrogate for sensitivity to anoikis. The time-course change in caspase-8 cleavage showed that a maximal cleavage was observed at 48 hours after onset of anoikis stress in the WiDr mock cells (Figure 1C). The level of the cleavage products decreased after that; however, the anoikis-related stress was still persistent and accumulated. In contrast to the mock, WiDr:MGAT5 became significantly resistant to anoikis: caspase-8 activation was almost completely suppressed during anoikis stress for up to 72 hours. The acquisition of anoikis resistance by MGAT5 overexpression was confirmed by immunofluorescence (Figure 1D). We also used conventional Annexin V/Propidium iodide (PI) protocol to take a closer look at the characteristics underlying the anoikis-related death. Mock cells were vulnerable to anoikis stress, and more than half of the exposed cells exhibited the early or late death phenotype. They were observed to predominantly follow the early apoptotic pathway (Figure 1E). The WiDr:MGAT5 cells appeared to experience late apoptotic death instead; however, the affected ratio was significantly lower than that of the mock cells.

The MGAT5-stimulated anoikis resistance was validated in clinical specimens by correlating the MGAT5 mRNA levels to the cancer stage. When the mRNA levels for MGAT5 were compared between cancer groups with a lower Astler-Coller grade (A-B2) and a higher grade (C1-D), elevated MGAT5 expressions were observed in colon cancer tissues of late stages compared to those of early stages (Figure 1F). The baseline information is listed in Supplementary Table 2. This may be explained by an interpretation that the metastatic cells undergo anoikis stress prior to completion of metastasis and that the MGAT5 upregulation is essential for the acquisition of anoikis resistance. Taken together, these data suggest that MGAT5 reinforces anoikis resistance of colon cancer cells.

### MGAT5 potentiates anchorage-dependent and -independent growth following anoikis stress

We tested whether the MGAT5-stimulated anoikis resistance culminates in enhanced cancer cell growth under varying conditions. The XTT-based viability test revealed that *ca*. 20% of the WiDr cells exposed to 48h-anoikis stress retained viability (Figure 2A). However, MGAT5 overexpression significantly rescued cells from anoikis with ≥50% of the stressed cells remaining viable. We then investigated anoikis time-dependent cell viability with an independent method (Figure 2B). Cells were subjected to anoikis stress for various times and then sub-cultured onto a new culture plate at a low density. Anoikis-resistant cells were quantified by the number of colonies formed on the culture plates. Mock cells showed a dramatic and continuous decrease in viability during anoikis stress. In agreement with the result in Figure 2A, an average of *ca*. 20% of the cells remained resistant to anoikis during the prolonged stress. However, the WiDr:MGAT5 cells remained viable for about 36 hours and showed decreased viability after that. These results suggest that MGAT5 confers survival advantages particularly in the early phase of anoikis-related cell death.

Next, we checked whether the anoikis resistance gained by MGAT5 overexpression favors cancer cell proliferation under anchorage-dependent as well as -independent conditions. Cells exposed to anoikis stress for 48 hours were embedded into soft agar (Figure 2C) or basement membrane-based matrix (Figure 2D), and their sphere-forming capabilities were investigated. It appeared that MGAT5 expression *per se* has a stimulatory effect on proliferation under anchorage-null conditions (Figure 2C). Such effects, *albeit* marginal, were also observed in the colonies grown in the basement membrane matrix (Figure 2D). However, the effect of MGAT5 expression on cancer cell proliferation was much more dramatic for anoikis cells under both anchorage-dependent and -independent conditions. The MGAT5 expression also caused an increased diameter of tumor spheres, which was more pronounced in the soft-agar beds. Collectively, these results indicate that MGAT5 confers survival advantages to cancer cells in an anchorage-dependent and -independent manner that would otherwise undergo apoptotic death following anoikis stress.

### Suppressed MGAT5 expression potentiates anoikis-induced apoptotic death

**Figure 3:**
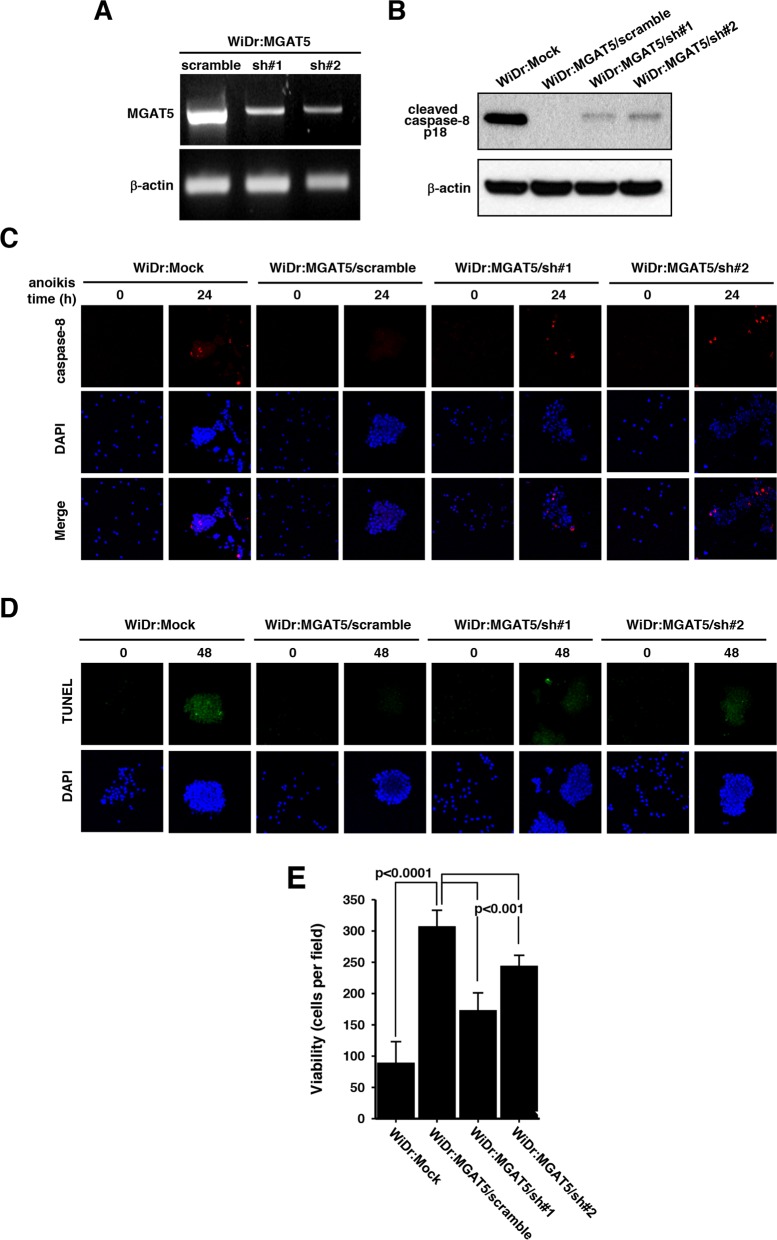
Sensitization of anoikis by suppressed MGAT5 expression **A**. The MGAT5 expression was suppressed through a small hairpin RNAs. The mRNA levels for two shRNA colonies were checked by RT-PCR followed on an agarose gel. **B**. Caspase-8 activation was compared among the mock, scramble, and MGAT5-suppressed cells by monitoring p18 products on an immunoblot. **C-D**. The apoptotic molecular signatures were monitored by caspase-8 activation through immunofluorescence **(C)** and by DNA fragmentation through TUNEL assay **(D)** following anoikis stress for 24-48 hours. **E**. Viability tests were performed by counting colonies formed on culture plates after anoikis stress for 48 hours. The values were averages of the number of colonies at 10 microscopic fields randomly selected at magnification of 400× (n=3).

To validate the effect of MGAT5 on resistance against anoikis, we established two transfectant cell lines with stable expressions of a small hairpin RNA (shRNA) for MGAT5. The MGAT5 expression level was down-regulated by the interference RNA confirmed by RT-PCR analysis (Figure 3A). The WiDr:MGAT5 cells with a scrambled shRNA expression did not show changes in caspase-8 activation. However, down-regulation of MGAT5 expression rescued the caspase-8 cleavage signatures (Figure 3B). The apoptotic molecular signatures were also monitored by immunofluorescence. Caspase-8 cleavage that disappeared by MGAT5 overexpression was rescued by the interference of MGAT5 expression (Figure 3C). The TUNEL assay also revealed the involvement of MGAT5 in the anoikis resistance (Figure 3D). The suppressed MGAT5 expression was connected to decreased anoikis resistance (Figure 3E). Taken together, the interference RNA-based approach enabled us to confirm that the viability of anokis-exposed cancer cells is critically enhanced by the MGAT5 expression level.

**Figure 4:**
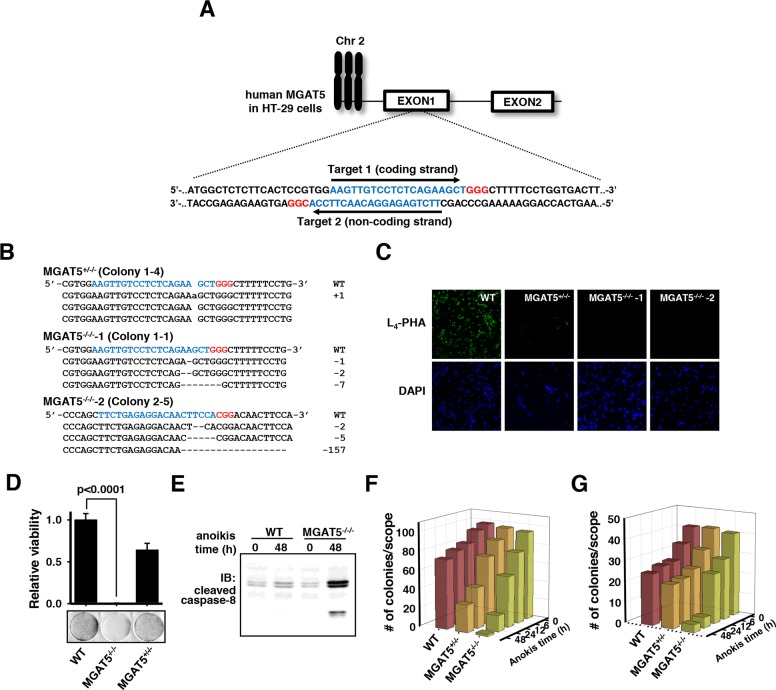
Validation of loss of anoikis resistance by MGAT5 gene knock-out in HT-29 cells using CRISPR/Cas9 **A**. Two targets for sgRNA were selected at the first exon of the MGAT5 gene. Target and PAM sequences are noted in blue and red letters, respectively. **B**. Gene editing using CRISPR/Cas9 created one heterologous and two homologous MGAT5 knock-out colonies. The indel mutations with a frame shift were confirmed by Sanger sequencing. **C**. Knockout of MGAT5 was confirmed by immunofluorescence using biotin-labeled L_4_-PHA and FITC-labeled streptavidin. **D**. The viability tests were conducted by imposing anoikis stress for 48 hours and then counting the colonies formed on culture plates (n=3). **E**. Caspase-8 cleavage was monitored by immuoblot analysis. **F-G**. Changes in the colony-forming capabilities by MGAT5 knockout were monitored under anchorage-dependent **(F)** and -independent conditions **(G)**. Cells were treated with anoikis stress for indicated times and embedded into Matrigels **(F)** and soft agars **(G)**.

To gain insights into the roles of the glycan structures in MGAT5-induced anoikis resistance, knock-out of the MGAT5 gene was performed in a different colon cancer cell line HT-29 by using the CRISPR/Cas9 system [23]. Both strands in the first exon of the MGAT5 gene were targeted (Figure 4A). Each target site was followed by the PAM sequence for recognition by Cas9. The transfection efficiency was assessed by fluorescence emitted by GFP fused to the N-terminus of Cas9, estimated to be in the range of 60-80% (data not shown). Ten colonies per single-guide RNA (sgRNA) were picked and subjected to T7E1 enzyme reactions. Colonies showing digestion with the T7E1 enzyme were analyzed for indel mutations by DNA sequencing, and one heterologous and two homozygous knockout cell lines were obtained (Figure 4B). The DNA sequencing data revealed the existence of the MGAT5 gene-located chromosome 2 as a trisomy in HT-29 cells [24]. The indel mutations invariably induced the early occurrence of nonsense mutations. These colonies were subjected to binding tests with phytohemagglutinin-L_4_ (L_4_-PHA) as a probe (Figure 4C). The immunofluorescence results confirmed that the homozygous knockout cells completely lost β1-6-N-acetylglucosamine (GlcNAc) glycan linkages on the cell surface.

The deletion of the MGAT5 gene almost completely attenuated anoikis resistance after exposure to anoikis stress for 48 hours compared to the wild-type cells (Figure 4D). The heterologous knockout cells were also more vulnerable to anoikis stress compared to the wild-type but retained a certain extent of resistance. These differences in the anoikis resistance level were observed by the apoptotic molecular signature: the caspase-8 cleavage increased dramatically even from a short duration of anoikis stress in the MGAT5 gene knock-out cells (Figure 4E). The anoikis time-course viability tests also confirmed that the MGAT5 knockout was responsible for the loss of anchorage-dependent (Figure 4F) as well as -independent (Figure 4G) sphere-forming capabilities. Collectively, MGAT5 activity had a pivotal role in anoikis resistance and thus we thought that intervention in the MGAT5 activity or its products could be a target to control anoikis resistance.

### Glycan profiling identified unique N-glycan structural features involved in anoikis resistance

**Figure 5:**
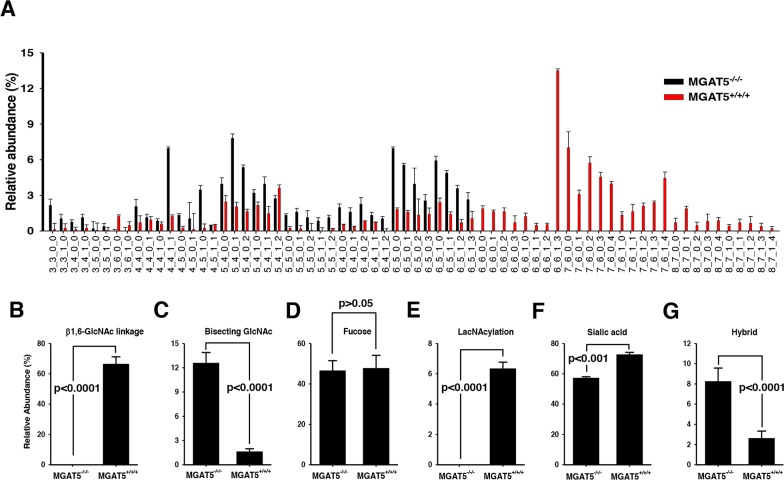
Comparison of N-glycan compositions for wildtype and the MGAT5 knock-out HT-29 cells **A**. The abundance ratios of each N-glycan component versus total N-glycans were compared between wildtype and MGAT5-knockout cells. Refer to the Supplementary Figure 1 for glycan structure corresponding to each code (n=3). **B-G**. The relative abundances of several N-glycan types were compared in wildtype and the MGAT5 knockout HT-29 cells. The types of N-glycans investigated were tetra-antennary β1,6-N-acetylglucosamine **(B)**, bisecting β1,4-GlcNAc **(C)**, core-fucosylated glycans **(D)**, poly-lacNAcylated glycans **(E)**, sialylated glycans **(F)**, and hybrid types **(G)**. P-values for all comparisons were <0.001 except for core-fucosylated glycans (n=3).

The upregulation of MGAT5 expression implies that tumor cells may be armored with altered glycan conformations and that MGAT5 has a critical role in the formation of such pro-metastatic glycan alterations. In fact, cumulative evidence supports this notion that there are profound alterations in the glycan structures and networks during the entire cancer cycle [25]. To take a closer look into the glycan-structural features that are obtained by MGAT5 expression, glycan-profiling analyses were conducted by comparing the relative abundances of an individual N-glycan between wild-type and the MGAT5-knockout HT-29 cells. Each N-glycan was resolved and identified by nano-LC/MS analysis [26], and the relative abundances were obtained by integrating the ion counts associated with each peak and normalizing to the total ion counts of all glycans. The relative abundances of each glycan code are compiled in Figure 5A, and the glycan structures for each code are shown in Supplementary Figure 1.

The N-glycans were grouped into several categories including high-mannose, fucose, sialic acid, poly-lacNAcylation, and bisecting GlcNAc-containing types. Afterward, their relative abundances were compared. As expected, the MGAT5-knockout cells showed no tetra-antennary N-glycan structures (Figure 5B). Instead, the bisecting GlcNAc level significantly increased (Figure 5C) [27]. In contrast, there were no significant fluctuations in fucose-containing N-glycans (Figure 5D). Features of interests were a high prevalence of poly-lacNAcylation (Figure 5E) and sialic acid-decorated branches (Figure 5F) and a low level of hybrid types (Figure 5G). To sum up, MGAT5 activity likely affects the levels of polylactosamine, bisecting GlcNAc, hybrid and sialic acid, and β1,6-GlcNAc branch.

### SSA treatment sensitizes anoikis-induced cancer cell death *in vitro* and *in vivo*

We hypothesized that one or more of these glycan features may be responsible for the recognition and transduction of the anoikis-resistance signals and that lectins with binding specificity to the corresponding glycan structures may be used to mask the signaling associated with anoikis resistance. To test this hypothesis, cells were subjected to anoikis stress in the presence of various lectins, each of which is known to have a binding specificity toward the glycan structures mentioned in Figure 5.

**Figure 6:**
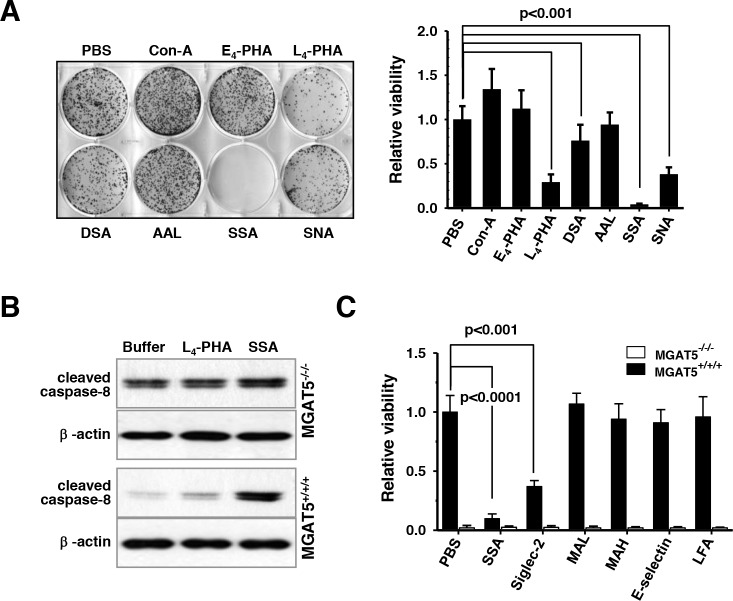
Sensitization of anoikis-induced cell death *in vitro* by SSA treatments under buoyant states **A**. Cells were anoikis-stressed for 48 hours in the presence of various lectins at 50 μg/ml. Then, cells were allowed to form colonies on culture plates and the number of colonies formed were counted (n=3). **B**. The lectin-based sensitization of caspase-8 activation was monitored by immunoblot analyses. **C**. Cell viability following 48h-anoikis in the presence of several sialic acid-binding lectins was investigated. Each value for MGAT5^−/−/−^ was considered non-significant (n=3).

As expected, the treatment with L_4_-PHA effectively sensitized the anoikis stress. The treatments with wheat germ agglutinin (WGA), Concanavalin A (Con-A), or *Aleuria aurantia* lectin (AAL) showed no noticeable effects; however, treatment with a lectin from *Datura stramonium* (DSA) was slightly, if not more, effective than that of L_4_-PHA (Figure 6A). A striking result was, however, observed for the use of sialic-acid binding lectins, SSA and *Sambucus Nigra* lectin (SNA). In particular, anoikis resistance acquired by MGAT5 overexpression almost completely disappeared in the presence of SSA. SNA attenuated the anoikis resistance, but the degree of inhibition was not as dramatic as that of SSA. The concentration-dependence tests revealed that a low concentration of SSA attenuated the anoikis resistance, and 10 μg/mL of SSA was enough to nullify the anoikis resistance by MGAT5 (Supplementary Figure 2). It is worth noting that the treatment of L_4_-PHA did not affect the anoikis resistance in the MGAT5-knockout cells indicating that the ß1,6-GlcNAc linkage is involved in the acquisition of the anoikis resistance (Supplementary Figure 2). SSA was inhibitory against anoikis resistance in the MGAT5-knockout cells, but the anoikis resistance of these cells was only marginal, suggesting that the sialic acid terminally attached to the tetra-antennary N-glycans, possibly bridged with the polylactosamine chain, may have a critical role in the acquisition of anoikis stress. The molecular signature also indicated that SSA and L_4_-PHA sensitized the anoikis-related apoptosis in a tetra-antennary N-glycan-dependent manner (Figure 6B).

Because SNA and SSA were effective in the sensitization of anoikis stress, additional lectins with sialic acid-binding specificity including *Maackia amurensis* lectin (MAL), *Maackia amurensis* hemagglutinin (MAH), *Limax flavus* lectin (LFA), sialic acid-binding Ig-like lectins-2 (siglec-2) and E-selectin were tested (Figure 6C). The anoikis-sensitization effect of SSA was unsurpassed by the other lectins. MAL and MAH, which were known to have a binding specificity toward sialic acid of α2,3-linkage [28], showed no effects on anoikis regulation. E-selectin, which has a specificity toward Lewis structures with sialic acid of α2,3-linkages, was also marginally effective. LFA does not have any specificity preference, and showed any notable effectiveness. However, lectins with an α2,6-linkages specificity, SSA and siglec-2 [29] showed a noticeable effects. These results indicate that use of lectins with an α2,6-linkages specificity can be used to control the anoikis resistance of cancer cells. It is not clear for now why SSA is especially effective compared to siglec-2 and SNA; however, it is believed that the MGAT5-initiated N-glycan branch has a favorable affinity toward SSA, compared to other sialic acid-binding lectins. It is noteworthy to mention that SSA can be used to enrich circulating tumor cells that show cancer stem cell-like features [30]. These results suggest that a tetra-antennary N-glycan structure with a terminal α2,6-sialic acid is a sufficient factor for anoikis resistance, and SSA effectively attenuates the MGAT5-induced anoikis resistance.

**Figure 7:**
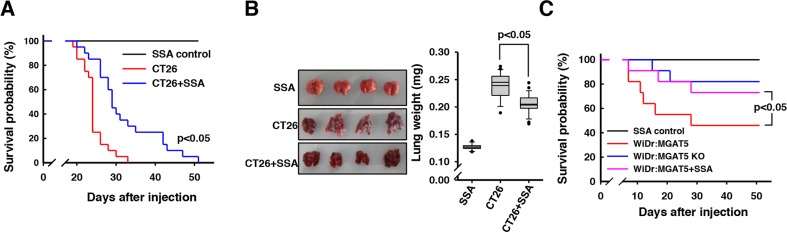
Enhanced survival of CT26 cells-injected mice by SSA co-injection **A**. A Kaplan-Meier survival analyses revealed a survival gain in the mice that were intravenously injected with 2×10^5^ CT-26 cells and co-injected with SSA (50 μg/ml) compared to those of buffer controls (n=20). **B**. A significant increase in the lung mass was observed for the CT26 cell-injected mice. SSA treatment significantly mitigated the increase in lung mass. **C**. The enhanced survival rates by SSA co-injection were confirmed for WiDr cells-injected mice (n=10).

The SSA-induced sensitization of anoikis stress was investigated *in vivo* with nude mice. CT26 is a metastatic mouse colon cancer cell and, when injected intravenously, results in pulmonary metastasis [31]. Based on the results *in vitro*, we investigated whether co-injection with SSA could serve as an agent to inhibit the hematogenous metastatic events during circulation. The tumor-injected mice began to die from *ca*. 20 days after injection. All mice (n=20) died within 33 days after injection; however, the survival rate was significantly enhanced by SSA co-injection (*p*<0.05) (Figure 7A). There was an almost two-fold increase in the lung weight with amorphous shapes in the CT26 cells-injected mice because of the tumor mass (Figure 7B). However, the SSA co-injection served to mitigate the lung weight gain by 19.2 % and the amorphous shapes of the lungs.

These results were confirmed with similar experiments using WiDr and mutant cells. The WiDr cells showed a milder metastatic phenotype compared to CT26 cells, and their metastatic sites were observed in the head, neck, and other organs, not confined to the lung. However, the survival rates were higher than the case of CT26. MGAT5 overexpression identically resulted in decreased survival rates; however, the SSA treatments enhanced the survival rate up to the level of the MGAT5-knockout cells (Figure 7C). These results clearly indicate that MGAT5 increases anoikis resistance of cancer cells during metastatic events, and SSA can be used as an agent to treat cancer patients with metastatic signatures. In particular, it could be used in patients who have circulating tumor cells in their blood.

## DISCUSSION

Cancer metastasis is a multi-step process where a variety of genetic machinery is employed to favor survival and translocation of cancer cells. Anoikis is considered to be a major hurdle for cancer cells to form a colony in remote tissues following detachment from the original site [5]. Death burdens begin to accumulate immediately after solid tumor cells lose adhesions to adjacent cells and extracellular matrices [22, 32]. Saturated amounts of these burdens lead to cell death. Accordingly, it is necessary to acquire anoikis resistance for tumor cells to achieve metastasis. For now, it is quite elusive how cancer cells acquire resistance against anoikis. Herein, we report how MGAT5 confers resistance to anoikis stress in colon cancer cells. MGAT5 triggered the formation of pro-metastatic N-glycan alterations [33], and α2,6-sialic acid linkages were critical for the acquisition of anoikis resistance. Importantly, the SSA treatments effectively controlled the anoikis resistance.

MGAT5 is considered to be an upstream regulator that affects many target proteins to promote cancer metastasis including MT1-MMP [19] and TIMP-1 [17, 18]. In addition, protein tyrosine phosphorylase kappa [34], matriptase [35], β_1_ integrin [36], and N-cadherin [37] are reportedly implicated in tumor progression either by direct or indirect mechanisms. The latter is best explainable by the galectin/glycoprotein lattice maintained by MGAT5 [38]. In these regards, identifying the target proteins of MGAT5 and understanding the molecular mechanisms behind the interplay between MGAT5 and its target proteins are necessary to establish the best strategy to control anoikis resistance in cancer cells. Because treatment with L_4_-PHA and SSA accelerated the anoikis-triggered death of colon cancer cells (Figure 6A–6C), the glycan branch carrying the β1,6-N-acetylglucosamine linkage of a particular target protein(s) may have a critical role in generating the anoikis resistance cues through a signal transduction pathway. Thus, elucidation of the molecular pathway is relevant to finding therapeutic options especially for patients with advanced cancer, which awaits further investigation.

Cancer metastasis accounts for the majority of deaths in many types of cancer. Nonetheless, there is no efficient treatment for patients with metastatic cancer. Cancer metastasis is necessarily accompanied by the penetration of cancer cells into the circulatory system, where metastatic cancer cells undergo anoikis stress during circulation. Anoikis is thus a critical barrier to successful metastasis, and many of cancer cells show apoptotic death in this stage. To overcome this challenge, cancer cells develop a variety of molecular machinery to survive anoikis stress. In fact, circulating tumor cells shows stem cell-like phenotypes and anoikis resistance [38, 39]. Furthermore, the number of circulating tumor cells in peripheral blood is associated with prognostic factors and disease-free survival [40, 41]. These studies stress the importance and clinical validity of the development of treatment options targeting anoikis. Because SSA was demonstrated to efficiently decrease MGAT5-triggered anoikis resistance *in vivo* as well as *in vivo*, it is highly plausible that SSA can be developed as therapeutics, as itself or an engineered form, for cancer patients who have anoikis-resistant, circulating or dormant tumor cells in the circulatory system.

## MATERIALS AND METHODS

### Treatment and measurement of anoikis *in vitro*

Colon cancer cell lines provided from the Korean Cell Line Bank (KCLB) were cultured in RPMI-1640 medium, supplemented with 10% FBS and 1% antibiotic solution at 37°C in 5% CO_2_ incubator. Cells were trypsinized, washed with PBS, and counted with an ADAM-MC cell counter (NanoEnTek). Anoikis stress was imposed by culturing cancer cells buoyant atop 3.5% soft agar-coated plates for up to 72 hours. After anoikis stress, cells were disaggregated into single cells in the Accumax solution (Innovative Cell Technologies), and were subjected to colony formation on culture plates or inside Matrigel (BD Biosciences) and 0.3% soft agar. While growing in in the solidified matrices, cells were supplemented with RPMI media containing 10% FBS. After cells were allowed to form sphere for up to 14 days, the size and number of colony were periodically measured on a microscope.

### DNA microarray

For control and test RNAs, the synthesis of target cRNA probes and hybridization were performed using Agilent's Low RNA Input Linear Amplification kit. The transcription master mix was also prepared as the manufacturer's protocol (4X Transcription buffer, 0.1M DTT, NTP mix, 50% PEG, RNase-Out, Inorganic pyrophosphatase, T7-RNA polymerase, and Cyanine 3/5-CTP). Transcription of dsDNA was performed by adding the transcription master mix to the dsDNA samples and incubating at 40°C for 2 hours. Amplified and labeled cRNA was purified on RNase mini-column (Qiagen). After checking labeling efficiency, each 750 ng of cyanine 3-labeled and cyanine 5-labeled cRNA target were mixed and the fragmentation of cRNA was performed by adding 10X blocking agent and 25X fragmentation buffer and incubating at 60°C for 30min. The fragmented cRNA was resuspended with 2X hybridization buffer and directly pipetted onto assembled Agilent Human whole genome 44K microarray. The arrays hybridized at 65°C for 17 h using an Agilent Hybridization oven. After washing, the hybridization images were analyzed by Agilent DNA microarray Scanner and the data quantification was performed using Agilent Feature Extraction software 9.3.2.1. The average fluorescence intensity for each spot was calculated with local background subtracted. Data analyses were performed using GeneSpringGX 7.3.1. Genes were filtered with removing flag-out genes in each experiment. Intensity-dependent normalization was performed, where the ratio was reduced to the residual of the Lowess fit of the intensity vs. ratio curve. The averages of normalized ratios were calculated by dividing the average of normalized signal channel intensity by the average of normalized control channel intensity. Genes with more than 2.0-fold changes were selected and considered as significant.

### Reverse transcription-PCR

Colon cancer tissue samples were obtained from colorectal cancer patients at Our Lady of Mercy Hospital at The Catholic University of Korea (Inchon, Korea) with agreement to participate obtained from all subjects. Resected tissues were immediately frozen and stored in liquid nitrogen until used. Messenger RNAs were prepared using a MaxWell automated nucleic acid purification system (Promega). Equal amounts of RNA were used to synthesize cDNA by using SuperScript III system (Invitrogen). Gene-specific primer pairs with sequences of ATGCTTCTGCACTTTACCAT (forward), GTGGAGTTGGTTGAGTTTGT (reverse) and TGTGTATGGCAAAGTGGATA (forward), ACCATGGTTTTTCACGTAAC (reverse) was used to measure the MGAT5 expression levels in the quantitative analyses.

### Immunoblot analyses

Trypsinized cells were washed with PBS, lysed in an extraction buffer [50 mM Tris-HCl, pH 7.4, 150 mM NaCl, 1% (v/v) NP-40] and centrifuged at 16,000 x g for 20 min. Protein concentrations of the lysates were determined using the Bio-Rad protein assay kit (Bio-Rad Laboratories). Proteins were resolved on SDS-PAGE gels and transferred electrically onto PVDF membranes (Millipore). The membranes were blocked in 0.05% Tween 20-TBS plus 5% skim milk and then incubated with an anti-cleaved caspase-8 (Cell Signaling) and anti-β-actin antibody (Sigma). After incubation with HRP-labeled secondary antibodies (Cell Signaling), membranes were allowed to react with ECL™ reagents (GE Healthcare) and exposed to X-ray film for 1-2 min.

### Flow cytometry analysis

For measurement of apoptotic signatures, cells were washed three times with cold PBS and then resuspended in binding buffer (10 mM HEPES/NaOH, 140 mM NaCl, 2.5 mM CaCl_2_, pH 7.4). Cells were incubated at room temperature for 15 min with annexin V-FITC and PI in the dark. Cells were sorted in a FACScan flow cytometer (Becton Dickinson).

### Immunofluorescence and tunel assay

Cells were fixed and permeabilized with BD Cytofix/Cytoperm™ solution (BD biosciences). Completely washed, fixed cells were incubated with an anti-cleaved caspase-8 antibody (Cell Signaling) for 2 h, washed with PBS buffer, and incubated with goat anti-rabbit immunoglobulin G antibody conjugated with an FITC fluorescent dye (SantaCruz) for 1 h. Cells were then washed with PBS buffer and stained with DAPI (Sigma). Tunel staining was performed using the fluorometric TUNEL staining kit (Promega Corporation). In brief, cells were fixed and permeabilized with BD Cytofix/Cytoperm™ solution (BD biosciences). After washing, cells were treated with 50 μl of recombinant fluorescein-12-dUTP cocktail for 1 h at 37°C in a humidified chamber. Cells were then placed onto a slide glass with mounting medium with DAPI and the slides were sealed. Cells were examined in an LSM510 Meta confocal fluorescence microscope (Zeiss).

### Construction of MGAT5 knock-out cell line

Pairs of oligonucleotides encoding the 20-nt sgRNA for MGAT5 were cloned into pSpCas9(BB)-2A-GFP (Addgene) plasmid vector. HT-29 cells were transfected with the plasmid vector using an Neon™ electroporator (Invitrogen). A single cell was seeded in 96-well plates and cultured to 80% confluency. Genomic DNA was isolated using a genomic DNA prep kit (Nanohelix) and used for subsequent PCR analysis and T7E1 assays. The final confirmation for knock-out was performed by the Sanger sequencing.

### Glycan profiling by LC/MS

N-glycans were released from cell membranes by incubating 100 μl of membrane samples with 2 μl of peptide N-glycosidase F (New England Biolabs) in 100 mM ammonium bicarbonate buffer (pH 7.5) at 37°C for 16 h. Then, proteins were removed by adding 800 μl of chilled ethanol, and the glycan samples were dried in vacuo. After reconstituted in PBS buffer, the glycan solutions were applied to a graphitized carbon cartridge (GCC). Then, the cartridge was washed with water at a flow rate of 1 ml/min to remove salts. Glycans were eluted stepwise with 10% ACN, 20% ACN, and 40% ACN, each of which contains 0.05% TFA. Each fraction was collected, dried in vacuo, and then reconstituted in water prior to MS analysis. GCC fractions were analyzed using a chip-based nano-LC/Q-TOF MS (Agilent). Separation was performed by a binary gradient A: 3% acetonitrile in 0.1% formic acid solution and B: 90% acetonitrile in 0.1% formic acid solution. The column was initially equilibrated and eluted with a flow rate of 0.3 μl/min for nano pump and 4 μl/min for capillary pump. The 65-min gradient was programmed as follows: 2.5-20 min, 0-16% B; 20-30 min, 16-44% B; 30-35 min, B increased to 100%, then continued 100% B to 45 min, finally 0% B for 20 min. Following chromatographic separation, glycans were ionized by a chip-integrated nano-ESI spray tip and analyzed by a 6530 Q-TOF mass analyzer. MS spectra were acquired in positive ionization mode over a mass range of m/z 500-2000 with an acquisition time of 1.5 seconds per spectrum. MS/MS spectra were acquired in positive ionization mode over a mass range of m/z 100-3000 with an acquisition time of 1.5 seconds per spectrum. Each possible composition of N-glycans was identified with the Molecular Feature Extractor algorithm included in the MassHunter Qualitative Analysis software (Version B.6.00 SP1, Agilent Technologies). The neutral monoisotopic mass of each compound was calculated using isotopic distribution, charge state information, and retention time. All ion signals associated with each compound were summed together to determine compound abundance. Computerized algorithms were used to identify N-glycan compositions by accurate mass. Deconvoluted experimental masses were compared against theoretical glycan masses using a mass error tolerance of 5 ppm.

### Tumor formation assay *in vivo*

All animal studies were performed in accordance with the guidelines of Institutional Animal Care and Use Committee after institutional review and approval. BALB/c-nude mice were obtained from Central Lab. Animal Inc. at approximately one month of age. Cancer cells were harvested using trypsin/EDTA solution and washed three times with PBS. Cells were suspended at 2 × 10^6^ cells per 100 μl of PBS and were injected into the tail vein of BALB/c-nude mice age-matched between 4-6 weeks. The viability of mice was daily checked and the lung tissues were immediately taken from mice for the measurement of masses.

### Statistical analysis

Data were obtained from at least three independent experiments and presented with averages and standard errors. Statistical differences between groups were determined by a Student's t-test. Values of p<0.05 were considered as significant. The Pearson coefficient was calculated using the Excel Pearson function and used to evaluate the correlation between transcription levels and anoikis resistance. For Kaplan–Meier plot analysis, two-tailed log-rank t test was used.

## SUPPLEMENTARY MATERIALS FIGURES AND TABLES






