# Supplementary material for: Lectin from Sambucus sieboldiana abrogates the anoikis resistance of colon cancer cells conferred by N-acetylglucosaminyltransferase V during hematogenous metastasis
Source: Oncotarget. 2017 Feb 2;8(26):42238–51. doi: 10.18632/oncotarget.15034 (PMC5522063; doi:10.18632/oncotarget.15034)
Supplement: Supplementary file 1 [file oncotarget-08-42238-s001.pdf]

Lectin from Sambucus sieboldiana abrogates the anoikis resistance of colon cancer cells conferred by N-acetylglucosaminyltransferase V during hematogenous metastasis

SUPPLEMENTARY MATERIALS

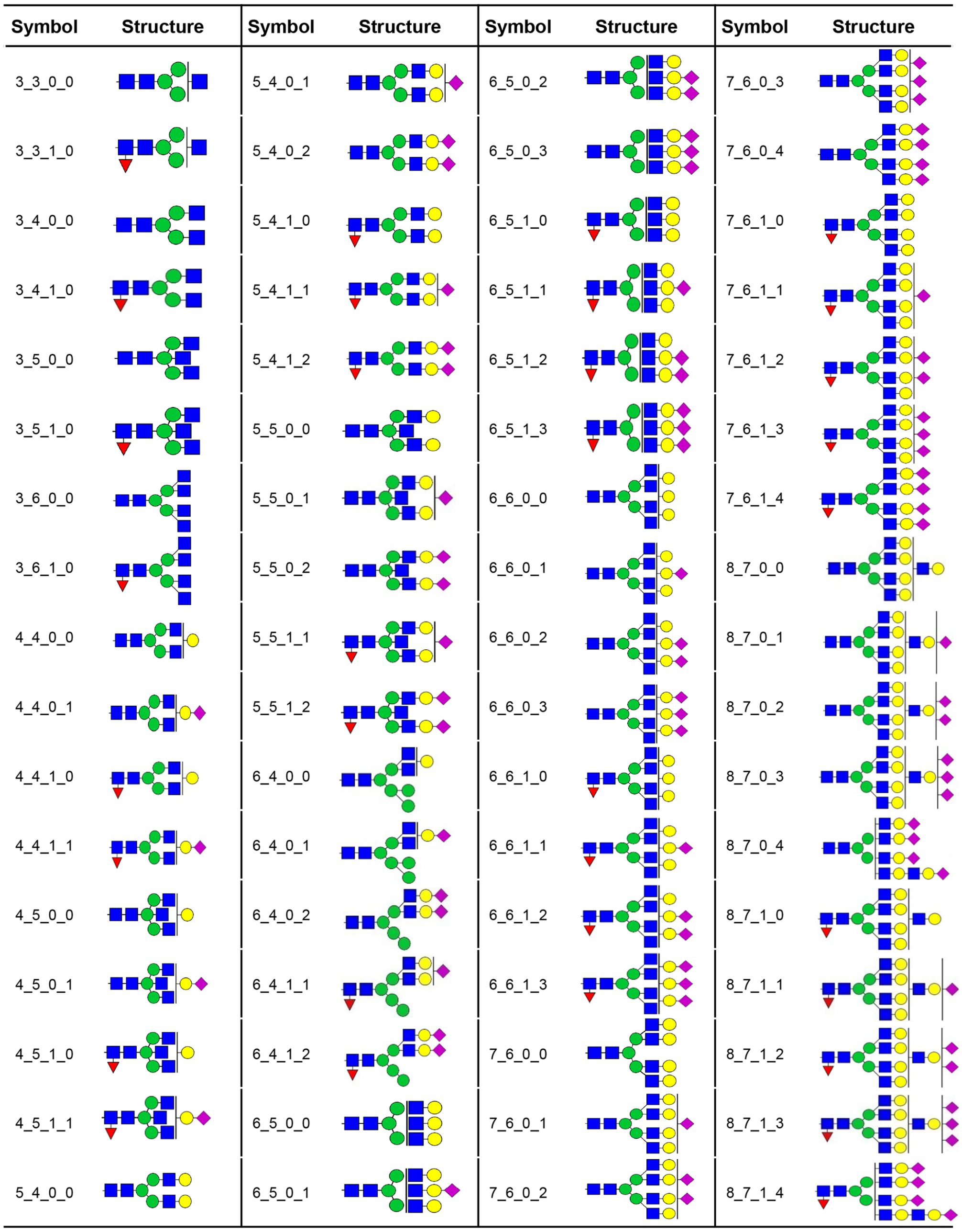

Supplementary Figure 1: Identified N-glycan structures with symbols. ■, N-acetylglucosamine; ●, mannose; ▼, fucose; ●, galactose; ♦, N-acetylneuraminic acid.

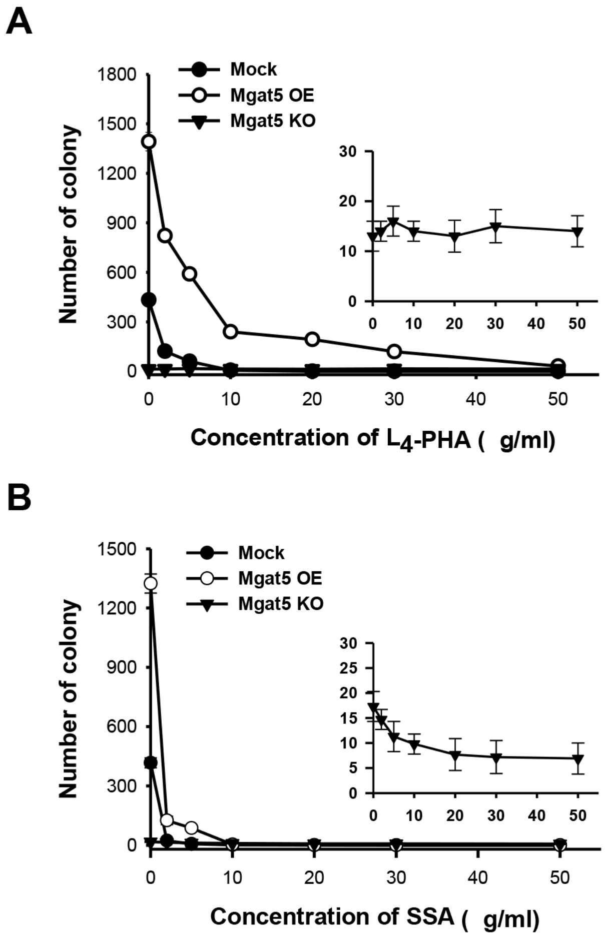

Supplementary Figure 2: Dependence of anoikis sensitivity on the concentration of L_4-PHA and SSA. HT-29 mock, Mgat5 overexpression transfectant (OE), and Mgat5 knock-out (KO) cells were exposed to anoikis stress for 48 hours in the presence of indicated concentration of L_4-PHA **A.** and SSA **B.** Cells were allowed to grow on culture plates for 3 days and the number of colony was counted on a microscope (X100). The average number of colony was compared between the HT-29 derivative cells. Inset shows the number of colony for Mga5 KO cells. The treatment L4-PHA did not affect the anoikis sensitivity, indicating that L_4-PHA binding and anoikis interference was specific to β1,6-GlcNAc branching. SSA treatment was slightly effective even for Mgat5 KO cells, which may suggest that sialic acids on N-glycan branches other than the β1,6-GlcNAc branch, albeit minutely, contribute to anoikis resistance.

Supplementary Table 1: Lists of genes correlated to anoikis resistance.

See Supplementary File 1

Supplementary Table 2: Clinical information of colon cancer patients.

See Supplementary File 2
